# A Warning to Dentists

**Published:** 1875-01

**Authors:** 


*A Warning to Dentists.*—A man giving his name as Anson A. Clark called upon Dr. Samuel Bridges, a dentist, Brooklyn, N. Y , Wednesday, and said that his wife wanted a set of teeth. He wished to surprise his wife, and would therefore pay the doctor in advance, directing him to give his wife a receipt in full when she called. Clark showed Dr. Bridges two checks, one for $100 and the other for $125, asking him if he would give the change. The doctor gave a check for $75, and the latter bade him good afternoon. A short time afterward the dentist became suspicious and went to the bank. He found that his check had been cashed, and that those of Clark were worthless.

